# Isolation of an escape-resistant SARS-CoV-2 neutralizing nanobody from a novel synthetic nanobody library

**DOI:** 10.3389/fimmu.2022.965446

**Published:** 2022-09-16

**Authors:** Dmitri Dormeshkin, Michail Shapira, Simon Dubovik, Anton Kavaleuski, Mikalai Katsin, Alexandr Migas, Alexander Meleshko, Sergei Semyonov

**Affiliations:** ^1^ Laboratory of Molecular Diagnostics and Biotechnology, Institute of Bioorganic Chemistry of the National academy of Sciences of Belarus, Minsk, Belarus; ^2^ Department of Biology, Belarusian State University, Minsk, Belarus; ^3^ Institute of Science and Technology Austria (ISTA), Klosterneuburg, Austria; ^4^ Imunovakcina, UAB, Vilnius, Lithuania; ^5^ Immunofusion, LLC, Minsk, Belarus; ^6^ Laboratory of Biosafety With Pathogens Collection, Republican Research and Practical Center for Epidemiology & Microbiology, Minsk, Belarus

**Keywords:** COVID-19, SARS-CoV-2, synthetic library, RBD, neutralization, nanobody, VHH

## Abstract

The COVID−19 pandemic not only resulted in a global crisis, but also accelerated vaccine development and antibody discovery. Herein we report a synthetic humanized VHH library development pipeline for nanomolar-range affinity VHH binders to SARS-CoV-2 variants of concern (VoC) receptor binding domains (RBD) isolation. Trinucleotide-based randomization of CDRs by Kunkel mutagenesis with the subsequent rolling-cycle amplification resulted in more than 10^11^ diverse phage display library in a manageable for a single person number of electroporation reactions. We identified a number of nanomolar-range affinity VHH binders to SARS-CoV-2 variants of concern (VoC) receptor binding domains (RBD) by screening a novel synthetic humanized antibody library. In order to explore the most robust and fast method for affinity improvement, we performed affinity maturation by CDR1 and CDR2 shuffling and avidity engineering by multivalent trimeric VHH fusion protein construction. As a result, H7-Fc and G12x3-Fc binders were developed with the affinities in nM and pM range respectively. Importantly, these affinities are weakly influenced by most of SARS-CoV-2 VoC mutations and they retain moderate binding to BA.4\5. The plaque reduction neutralization test (PRNT) resulted in IC50 = 100 ng\ml and 9.6 ng\ml for H7-Fc and G12x3-Fc antibodies, respectively, for the emerging Omicron BA.1 variant. Therefore, these VHH could expand the present landscape of SARS-CoV-2 neutralization binders with the therapeutic potential for present and future SARS-CoV-2 variants.

## Introduction

The severe acute respiratory syndrome coronavirus 2 (SARS-CoV-2) has caused a global pandemic with the number of coronavirus disease (COVID-19)-related deaths exceeding 5.6 million as of February 2022 (1). Despite the unprecedented success in the SARS-CoV-2 vaccine development, this number is still growing, raising the question of multiple antiviral therapeutics development.

The SARS-CoV-2 virus entry is initiated on the host cell surface by the attachment of spike (S) protein receptor-binding domain (RBD) to angiotensin converting enzyme 2 (ACE2). It became obvious that antibodies could prevent virus fusion with the cell membrane by blocking RBD-ACE2 interaction or fixing the RBD in “down” conformation (2, 3).

Therapeutic neutralizing antibodies constitute a key short-to-medium term approach to tackle COVID-19. The last outbreak of the Omicron (B.1.1529) variant abrogated the majority of the FDA-authorized antibody treatments (4). Despite the lower mortality rate from the Omicron variant, its antibody treatment is still in high demand, especially for immunosuppressed patients, as well as for high risk group patients who are not able to be fully vaccinated (5). In early April 2022, two new Omicron lineages were reported from South Africa and designated BA.4 and BA.5 (6). They became dominant due to the ability to avoid vaccine-raised antibodies and therapeutic monoclonal antibodies increasing the demand for new therapeutics development (7).

As the antibody discovery and clinical approval is a time-consuming process even under the special conditions for COVID-19 treatment authorization, it is necessary to have a rapid and robust pipeline of VoC neutralizing binders identification and isolation. Synthetic libraries and *in vitro* display selection processes proved themselves during the pandemic yielding a variety of SARS-CoV-2 antibodies of different origins and shapes in quick terms (3, 8, 9). One of the most promising classes of binders are nanobodies (VHH). Nanobodies have a number of remarkable features that facilitate their expansion as universal binders such asr small size, good stability and solubility, cryptic epitopes or cavities binding ability (10).

In the present work, the synthetic humanized nanobody library with a diversity of more than 10^11^ variants was developed. The resulted diversity in complementary-determining regions (CDR) mimics natural VHH diversity, but exceeds it in its size.

We report a pipeline for the generation of VHH-based neutralizing binders from the diverse humanized synthetic library. As a proof-of-work we identified an antibody G12 that binds Wuhan-Hu-1 (WT) and Delta (B.1.617.2) variants of SARS-CoV-2 with the two-digits nanomolar affinity constant. Utilizing affinity and avidity engineering we explored the possibility of therapeutic potential enhancement of this clone. We succeeded in generation of affinity maturated H7-Fc with 2-9 nM affinities range to VoCs, including Omicron (BA.1) and triple G12x3-Fc with the pM affinity to the Delta variant and 3 nM affinity against Omicron. We demonstrated that isolated antibodies possess strong binding and neutralization characteristics for highly transmissible Delta and Omicron variants, not inferior to the FDA approved (EUA) antibodies for COVID-19 treatment.

This selection strategy could be efficiently expanded towards new variants of SARS-CoV-2 in order to expand the currently existing landscape of VHH neutralizing binders with the therapeutic potential. The data suggest that H7-Fc and G12x3-Fc antibodies could be a promising therapeutic agent for COVID-19 treatment, which may retain efficacy against continuously developing variants.

## Material and methods

### Reagents and solutions

Sulfo-NHS-SS-Biotin, streptavidin-PolyHRP, Dynabeads T1, Tween-20, PEG8000, FreeStyle™ Expression Medium, Nunc Maxisorp high-binding plates were purchased from Thermo Fisher Scientific. TB, LB, 2xYT medium, PBS were bought from Melford Laboratories Ltd. M13KO7 phage helper, *ER2738* strain and all enzymes were purchased from New England Biolabs Inc. Other chemicals were purchased from Sigma and used without further purification and additional preparation. All solutions were prepared using deionized MilliQ quality water.

### SARS-CoV-2 virus variants

Three isolates of SARS-CoV-2 were used: 2245 (B.1.1.1), 2107 (B.1.617.2.122, Delta derivative), 1984 (B.1.1.529.1, Omicron variant). The isolates were obtained in 2021 and 2022 at the Biosafety laboratory of the Republican Scientific and Practical Center for Epidemiology and Microbiology (Republic of Belarus) from nasopharyngeal swabs of patients with symptoms of COVID-19 and typed using sequencing.

### Production of recombinant SARS-CoV-2 RBD variants and hACE2 protein

For the initial VHH selection experiments and binding affinity measurements a codon optimized RBD (residues 319 to 529) of spike protein from SARS-CoV-2/human/China/Wuhan-Hu-1/2019 (GenBank: QHD43416.1) and Omicron (IPBCAMS-OM01/2021) were generated by gene synthesis (Synbio-tech). Selected mutations for the generation of Beta B.1.351, Delta B.1.617.2 and Delta plus B.1.617.2/AY.1 variants were introduced into Wuhan-Hu-1 RBD by site-directed mutagenesis with the primers listed in **Table S2**. All the RBD variants were cloned into mammalian expression vector pcDNA3.2 (Thermo Fisher) with the additional N-terminal HA leader sequence (MNTQILVFALIAIIPTNADKIGSGA) and C-terminal 10x His tag. ACE2 gene was amplified from cDNA with the primers hACE2-f\hACE2-r and cloned into a custom modified pFUSE-hIg1d expression vector (Invitrogen). SARS-CoV-2 Spike RBD Omicron BA.4&BA.5 was purchased from Acro Biosystems (SPD-C522r, SPD-C522g).

Endotoxin-free purified plasmids were transiently transfected in FreeStyle™ 293-F cells. FreeStyle™ 293-F cells were passaged at 0.5 × 10^6^ cells/mL in 40 mL of fresh FreeStyle™ Expression Medium in 250 ml polycarbonate shake flask (Corning). The following day, 40 μg of DNA and 80 µg of polyethyleneimine (10 kDa, linear), separately diluted in 0.1 mL of Opti MEM (Gibco), were vigorously mixed. The mixture was incubated for 15 minutes at RT and then added to the cells. Transfected cells were incubated at 37°C, 8% CO2 for 5 days on an orbital shaker platform (125 rpm). Transfected supernatants were collected 5 days after expression, clarified with centrifugation (800 g, 5 min), filtered with a 0.2 µm PVDF filter and purified over HisPur and Protein A column for RBD and hACE2-Fc proteins, respectively, using ÄKTA Purifier 10 FPLC System (Cytiva Life Sciences). Both recombinant SARS-CoV-2 RBD and hACE2-FC were further purified to homogeneity using a Superdex 75 Increase 10/300 column (Cytiva Life Sciences).

The RBDs were biotinylated by amine coupling with sulfo-NHS-SS-Biotin according to the manufacturer’s manual (Thermo Fisher). Briefly, fresh sulfo-NHS-SS-Biotin (10 mg/mL in PBS; pH 7.4) was rapidly added to RBD in PBS at a molar ratio of 20:1. After 1 h of reaction at room temperature (RT) with gentle shaking, free biotin was removed through extensive dialysis at 4°C. Then biotinylated RBDs were stored in aliquots (at -80°C until use).

The full-size SARS-CoV-2 protein was purchased from the commercial vendor Invitrogen (#RP-87680).

### Synthetic VHH library generation

A VHH scaffold sequence h-NbBCII10_FGLA_ was codon optimized for *E. coli* and *H. sapience* expression and purchased from Synbio Technologies. TRIM based randomization oligos were also purchased from Synbio Technologies.

Kunkel mutagenesis and electrocompetent cells preparation were performed according to the Sidhu lab protocol (11). Purified CCC-dsDNA was used in RCA for template elimination and mutated DNA amplification (12). 20 µg of each CCC-dsDNA pool was divided between two reactions of 350 µl highly competent *E.coli ER2738* pre-infected with M13KO7 electroporation. Sub-libraries sizes were estimated by the titration.

### Identification of SARS-CoV-2 binders

A proprietary humanized synthetic VHH library with the diversity exceeding 10^11^ unique antibody clones was used as a source of RBD-specific binders. Two high-binding Nunc microplate wells were coated with hACE2-FC at 2 μg/ml in PBS overnight at 4°C (100 µl per well). The next day plate wells were blocked with 1% BSA in PBST for 1 h and 3 ug/ml RBD was added for 1 hour. At the same time, 20 µl of Dynabeads T1 streptavidin magnetic beads were blocked with 0.5% BSA for 1 h and then washed 3 times with 0.1% PBST (PBS supplemented with 0.1% Tween-20). VHH libraries were added to RBD-ACE2 complexes for 30 min and then were transferred to a 1.5 ml low protein-binding tube with 100 nM biotinylated RBD in 0.5% BSA. After 60 min of incubation, the phages/RBD mixture was transferred to streptavidin magnetic beads for 10 min. Then the tube was placed in a magnetic particles separator and allowed to stand for 30 s to remove the supernatant containing the unbound phages. The particles were washed 10 times by cycles of magnetic rack separation and resuspension in PBST. Finally, the phages were released by 30 min incubation with 100 mM DTT for -S-S- bond disruption and specific elution of RBD-bounded phages. Eluted phages were used for titration and amplification in *E. coli ER2738* for additional rounds of selection. In total, four rounds of selection were made. Selective pressure was maintained by RBD concentration decreasing (from 100 nM to 10 nM) and washing stringency increasing (from 10 times in the 1st round to 20 times in the 4th). In the last round, off-rate selection with the presence of 5 µM of non-biotinylated RBD was performed within 5 min. After the last round, individual clones from the titration plate were used for phage supernatants production by overnight cultivation in 200 µl of 2xYT medium, supplemented with 50 µg/ml kanamycin and 100 µg/ml carbenicillin, in 96-well cell culture plate at 30°C with continuous shaking. Cells were pelleted at 3200 g for 30 min. 20 µl of 10x PBS with 0.5% NaN_3_ was added to phage supernatants before storage at 4°C for phage ELISA.

### Phage monoclonal and competitive ELISA

For phage monoclonal ELISA 32 wells of a 96-well microtiter plate were coated with 2 µg/ml RBD (positive wells) in 100 µl PBS and 32 wells with 5 µg/ml BSA (negative wells) overnight at 4°C. Afterwards, the plate was blocked with 5% skimmed milk in PBS for 2 hours at RT. 5 µl of individual phage supernatants were mixed with 95 µl of 2.5% (w/v) skimmed milk. The mixtures were added to the wells and incubated at RT for 30 min. After incubation, the wells were washed 6 times with 0.1% PBST and 100 µL of anti-M13 phage antibody conjugated with HRP (1:5000 dilution in PBS) was added. After 1 h of incubation and 6 washes, positive binders were determined upon 100 µl TMB substrate addition. The absorbance at 450 nm was determined after stopping the reaction by adding 100 μL of 2 M H_2_SO_4_ per well. The top twenty binders, according to their signal-to-noise ratio, were subjected to sequencing and a competitive ELISA assay.

For the competitive phage ELISA, an RBD-coated plate was prepared as described above. Phages (10^9^ PFU/ml) in 100 µl of 2.5% skimmed milk in 0.05% PBST were incubated with 3 different concentrations of RBD (0 nM, 10 nM, 100 nM) for 1 hour at RT with continuous shaking. Then mixtures were transferred to RBD-coated wells for 15 min and washed six times with 0.1% PBST. Detection of bounded phages was done as described above for monoclonal ELISA.

### VHH affinity maturation and tandem repeats formation

For the affinity maturation of the G12 clone a FR1-CDR1-FR-2-CDR2 region was randomized by subcloning this region from pooled synthetic libraries phagemids into the G12 sequence. The resulted diversity was electroporated into *E.coli ER2738* pre-infected with M13KO7 phage for G12-based library construction as described elsewhere (11). Three rounds of biopanning were performed with different RBD concentration from 10 nM to 100 pM as described above for SARS-CoV-2 binders identification.

For multimeric tandem VHH construction G12 was amplified in 3 separate PCR reactions with primers pairs VHHtri-NcoI-f/VHHtri-BamHI-r, VHHtri-BamHI-f/VHHtri_XhoI-r and VHHtri-XhoI-f/VHHtri-NotI-r (**Table S1**). VHH fragments were digested by *NcoI\XhoI, XhoI\SalI, SalI\NotI* respectively and ligated simultaneously into *NcoI\NotI* digested into a custom modified pFUSE-hIg1 expression vector (Invitrogen).

### Production of VHHs in mammalian cells in fusion with human IgG1 Fc (VHH-Fcs)

The antibody sequences (monomeric and multimeric tandem repeats) were cloned into the pFUSE-hIg1d expression vector for transient transfection. pFUSE-hIg1d is a modified pFUSE-hIg1 (Invitrogen), in which *NotI* restriction site in the backbone was eliminated by site-directed mutagenesis and incorporated before the Fc domain. The Fc-fusion proteins were produced by transient transfection of FreeStyle™ 293-F cells followed by Protein A affinity chromatography as previously described for ACE2-Fc protein. Then Fc-fusion proteins were dialyzed against PBS and concentrated with Amicon Ultra-4 centrifugal unit (MWCO 30 kDa). Their purity and integrity were verified by reducing SDS-PAGE and MALDI-TOF MS.

### Bio-layer interferometry assay

The binding kinetics of antibodies to RBD was measured by BLI on an Octet-R2 (Sartorius). The his-tagged RBD variants were loaded onto Ni-NTA biosensors from 10 ug\ml solution in kinetic buffer (PBS, 0.02% tween-20, 0.05% BSA, filter-sterilized) for 300 s. The sensors were equilibrated (baseline) for 60 s, before incubating with VHH-Fc nanobody fusion proteins at various concentrations from 0.5 nM to 100 nM (association) for 200 s. Dissociation kinetics were measured by dipping sensor tips into wells containing kinetic buffer for 200 s. Data were reference subtracted (reference sensor and reference sample), and kinetics were calculated in Octet Analysis Studio v.12.2 using a 1:1 binding model.

### Plaque reduction neutralization test

Biosafety Level 3 laboratory setting was used for PRNT tests. For each test, serial ten-fold dilutions of samples starting at the concentration of 10 μg/ml were prepared in Dulbecco modified Eagle medium (DMEM). The dilutions, mixed to a 1:1 ratio with a virus solution containing about 30 plaque-forming units (PFU) of SARS-CoV-2 per well (previously determined by virus titration), were incubated for 1 h at 37°C. Sixty microliters of the virus-sample mixtures were added to confluent monolayers of Vero E6 cells in 96-well plates in triplicates and incubated for 1 h at 37°C in a 5% CO2 incubator. The inoculum was removed and 100 µl of overlay solution of DMEM, 2% fetal bovine serum (FBS), penicillin (100 U/ml), streptomycin (100 U/ml) and 1% carboxymethylcellulose were added to each well. After incubation at 37°C in a 5% CO2 atmosphere for 76 hours, cells were fixed with a 4% formaldehyde solution, stained with crystal violet (0.05%) and plaques were counted. IC50 value was calculated by logistic regression using an online tool “Quest Graph™ IC50 Calculator.” AAT Bioquest, Inc., 21 Apr. 2022, https://www.aatbio.com/tools/ic50-calculator.

### Library preparation for NGS

Phagemid DNA isolated from one of the unselected phage sub-libraries was used as a PCR template in order to amplify VHH and add the adaptors required for Illumina sequencing. Amplified VHH were purified from the agarose gel using GeneJET PCR Purification Kit (ThermoFisher scientific), and its concentration was adjusted to 80 ng\µl. The VHH library was then analyzed, quantified and sequenced using the Illumina MiSeq platform.

Data analysis was performed by the custom python 3.10-written algorithm. Codding frames were found by the Biopython package, and “Chotia” annotation was performed using ANARCI library which is available for free on GitHub (https://github.com/oxpig/ANARCI). All other data manipulation were handled by build-in python functions.

## Results

### Synthetic humanized nanobody library design and development

**Figure 1:**
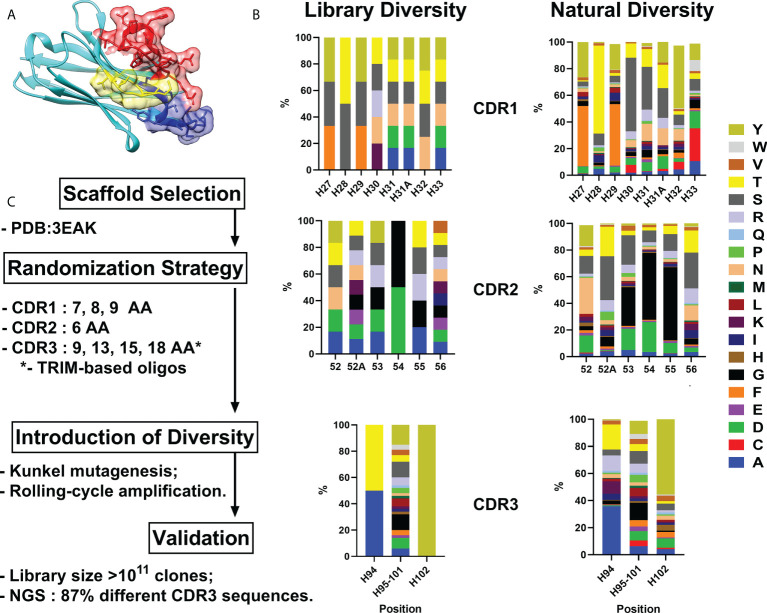
VHH library generation pipeline. Humanized VHH scaffold **(A)** – CDR1, CDR2 and CDR3 are depicted in blue, yellow and red respectively. **(B)** - Comparison between relative amino acid frequencies in CRD1, CDR2 and CDR3 observed from Camelid VHH sequences and VHH4.0_DD library. **(C)** – Library generation and validation scheme. Amino Acid are indicated in single letter code.

As all sequences in a synthetic library share more than 75% identity with the parental scaffold antibody, it is crucial to choose an appropriate one for randomization. We formulated a number of requirements that an ideal scaffold must possess as follows: 1) high expression yields in bacteria and mammalian systems; 2) available crystallographic data; 3) no freedom to operate restrictions for commercial use; 4) excellent CDR-grafting possibilities; 5) high thermal stability. Humanized variant h-NbBCII10_FGLA_ of nanobody NbBcII10 meets the requirements and was chosen as a scaffold for the library (**Figure 1A**) (13, 14).

For the randomization strategy, 650 *Camelidae* VHH sequences from abYsis database were analyzed for CDR’s lengths and amino acids composition (15). Trinucleotide oligos were designed to mimic natural diversity but with a lower proportion of hydrophobic residues and the absence of cysteines (**Figure 1B**).

Kunkel mutagenesis was performed for the diversity introduction. Four different Kunkel mutagenesis reactions (one sub-library for each CDR3 length) were carried out (**Figure S1**). 5 µg of CCC-dsDNA product after each reaction were subjected to rolling-cycle amplification (RCA) reaction resulting in amplification to 20 µg of CCC-dsDNA presumably with a lower rate of uracil incorporation and without parental scaffold VHH. Every CCC-dsDNA pool was electroporated into highly-electrocompetent *E.coli* ER2738 – two 10 µg electroporation reactions for every CCC-dsDNA pool. As a result, the synthetic humanized nanobodies library VHH4.0_DD consisted of 4 sub-libraries with a diversity of ~3×10^10^ clones each was constructed.

For validation purposes, 24 random clones were picked and sequenced by Sanger. All the clones had a different CDRs composition without any frame shifts or mutations in FR regions. In order to expand the scope of the analysis, NGS sequencing was performed. Out of all the reads that passed QC and cover CDR3 with the flanking regions (n=32 547), 28 027 were unique which gives >86% diversity of the library.

A comprehensive block diagram of the described library development is presented in **Figure 1C**.

### Identification of VHH against SARS-CoV-2

**Figure 2:**
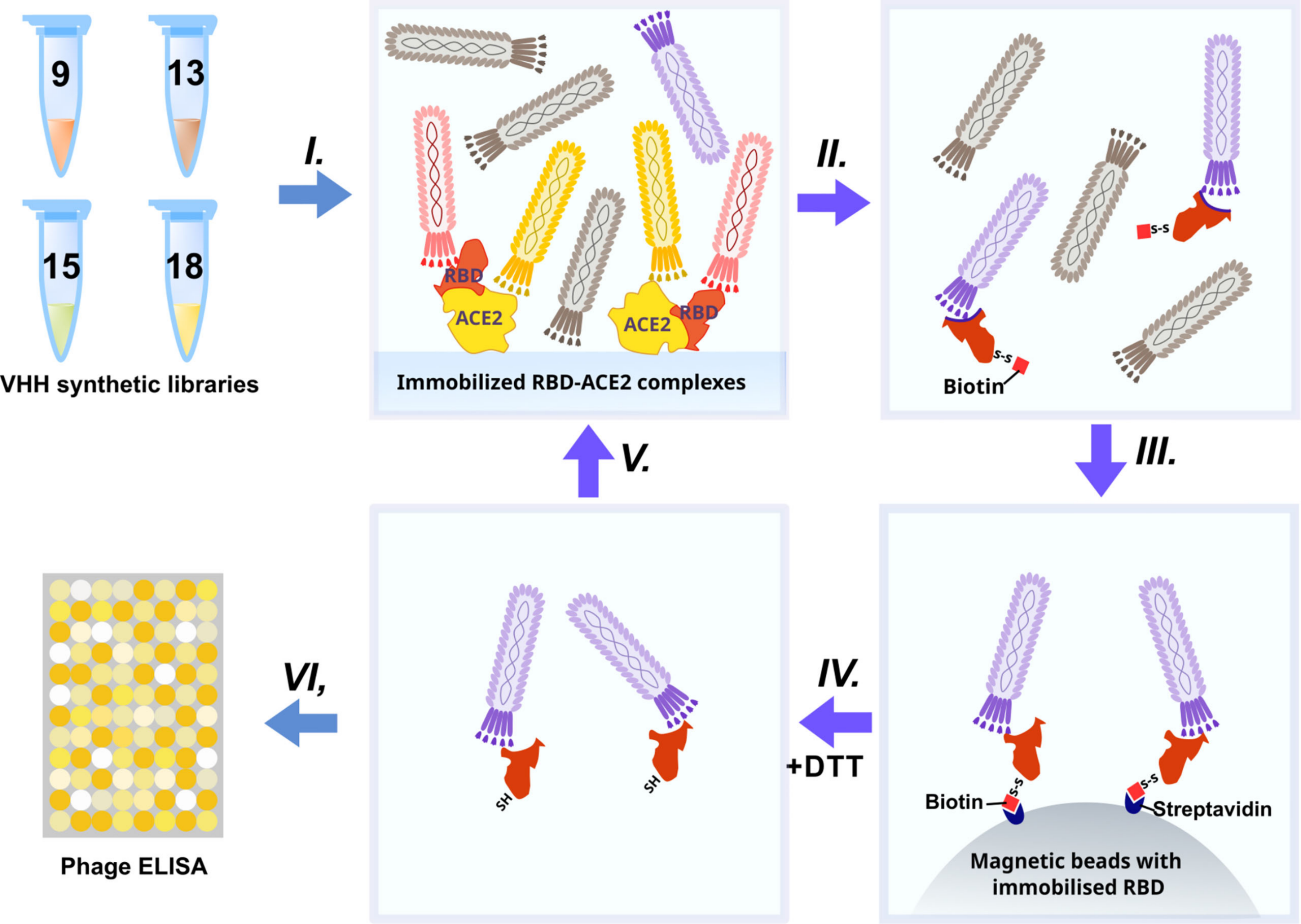
Schematic representation of RBD-binders phage display selection.

The synthetic humanized nanobody library VHH4.0_DD was used as a source of RBD binders. The VHH4.0_DD library proved its suitability and previously yielded a number of low-nanomolar binders to different targets, including difficult poly-glycosylated (16). In order to enrich the binders that block the interaction of RBD with the ACE2 receptor, we utilized a negative selection approach (**Figure 2**).

Briefly: the phage display library was incubated with the immobilized RBD-ACE2 complex (*I)*. Antibodies that bind the RBD-ACE2 interface mostly remained in the solution, while sticky and unspecific binders were depleted. Then the supernatant was transferred to an Eppendorf tube with the biotinylated RBD protein (*II*) (WT for the first round, Beta (B.1.351) for the second, Delta for the third, and Omicron BA.1 for the fourth) for 2 h incubation and pulled down with the streptavidin magnetic beads afterward *(III)*. As RBD proteins were chemically biotinylated with the -S-S- containing agent, mostly RBD-specific phages were eluted with DTT treatment from magnetic beads after pull down and washing *(IV)*. Off-rate selection during the last round ensured the selection of the tightest binders with the slowest dissociation rate (*k_off_*) and the highest affinity (17). The concentration of the RBD was sequentially reduced from 200 nM in the first round to 10 nM in the last round *(V)*.

**Figure 3:**
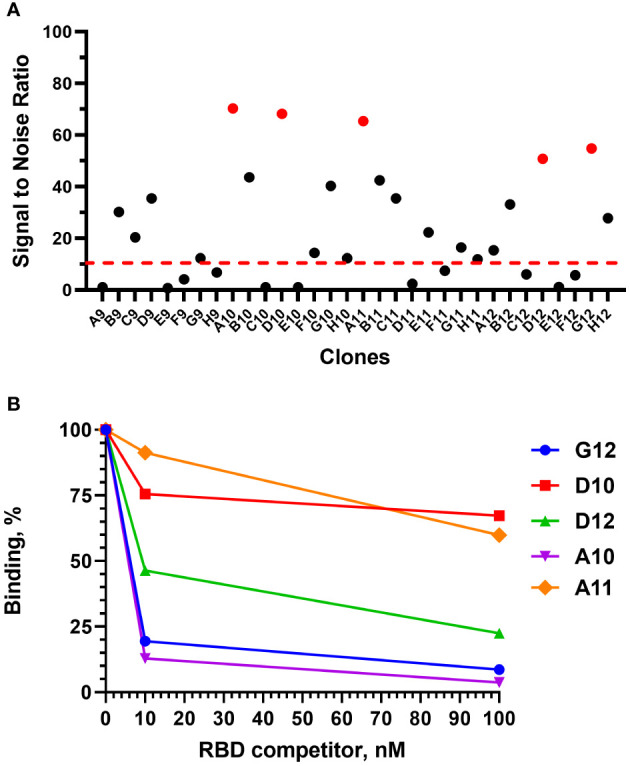
Monoclonal ELISA of selected VHH clones (pointed red) **(A)** and competitive phage ELISA **(B)**. Dashed red line – S\N threshold.

After 4 rounds of selection, 32 individual clones were analyzed by monoclonal ELISA (**Figure 3A**).

**Figure 4:**
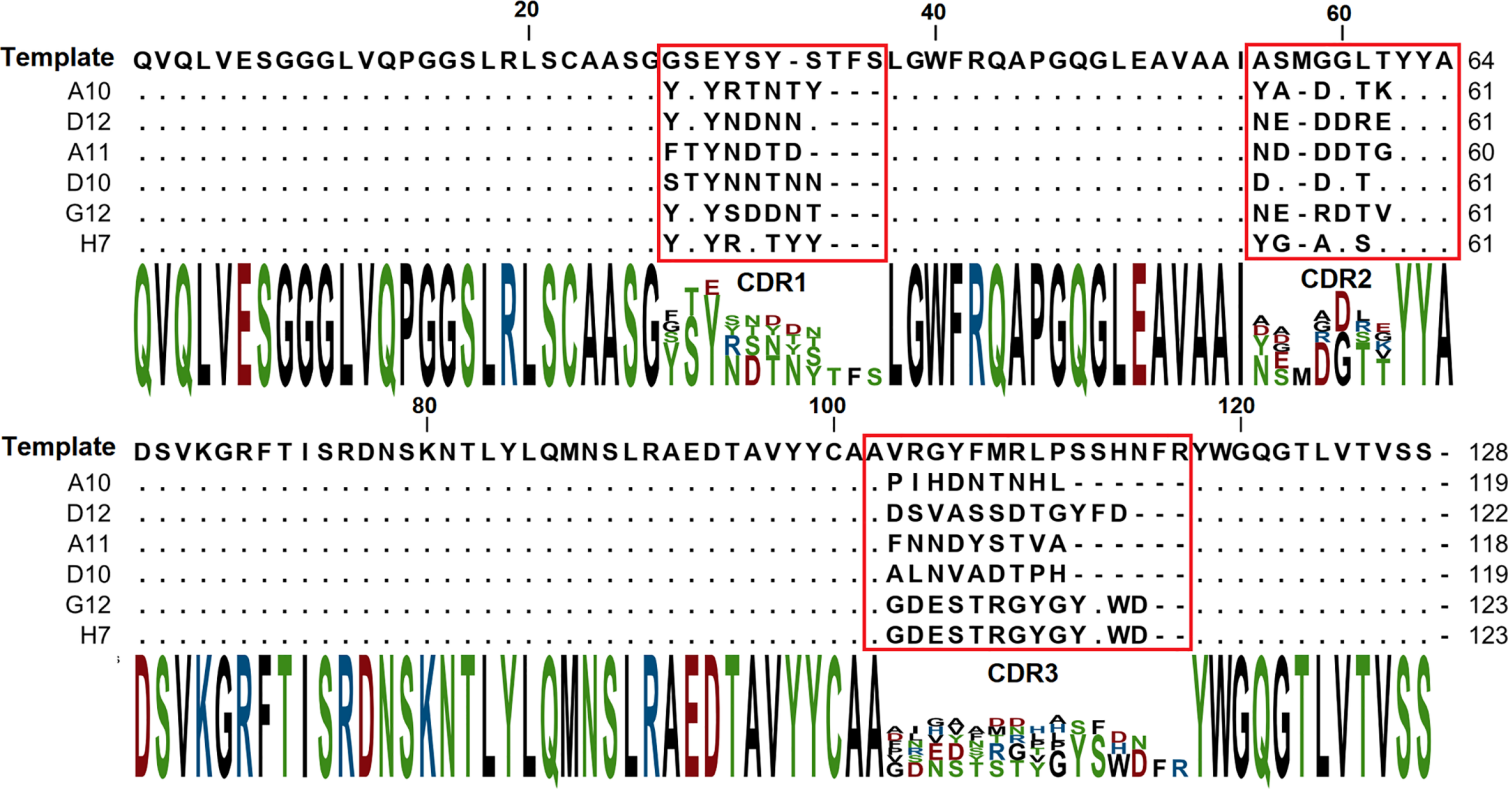
Amino acid sequences of selected VHH binders. CDR sequences are marked with red.

The binding of each clone to the RBD was compared with the binding to a BSA, resulting in a signal-to-noise ratio >10 for 20\32 clones. Binders with the highest signal-to-noise ratio in monoclonal ELISA (A10, D12, A11, D10, G12) were isolated and sequenced (**Figure 4**).

A competitive phage ELISA allowed us to range the top 5 binders by their *k*_off_ value and exclude binders that poorly bind soluble antigen in a liquid phase (**Figure 3B**). Antibodies with the fastest dissociation rate were recaptured by the immobilized antigen resulting in a high binding signal after anti-M13-HRP detection. The steepest signal decreasing with the increase of the antigen concentration (from 0 to 100 nM) corresponded to the binders with the slowest dissociation. Antibodies with the slowest dissociation rate are considered better binders.

**Figure 5:**
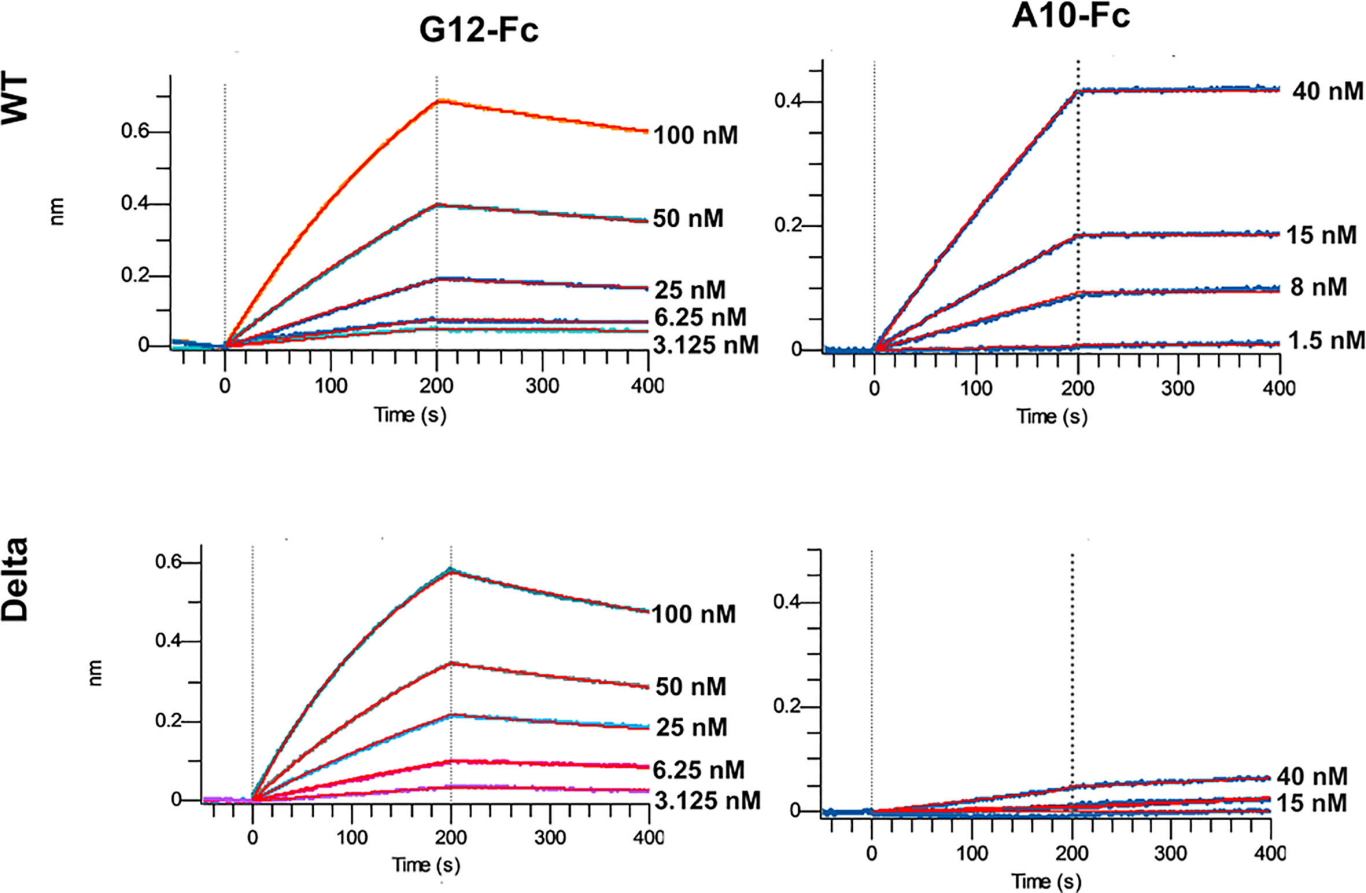
Multipoint Biolayer Interferometry (BLI) of VHH-Fc binders to WT and Delta variants RBD.

The most promising G12 and A10 binders were reformatted in the VHH-Fc fusion format for affinity and specificity determination against WT and Delta strains by means of biolayer interferometry with the use of Octet R2 (**Figure 5**).

The A10-Fc antibody possessed a very high binding affinity of 25 pM, but showed no significant binding to the Delta. Antibody G12-Fc had a lower affinity of 20 nM, but its binding was not affected by Delta variant’s mutations. It was recently demonstrated that low-nanomolar binders could exhibit excellent neutralization potency with a single digit IC50, ng\ml values (18). Nevertheless, in most cases there is a strong correlation between the affinity of the antibody and its neutralizing potential (3, 19). Two digits nM affinity of the G12-Fc allows it to be considered as a high-affinity binder, but it could not be sufficient for effective neutralization of SARS-CoV-2 infection with the IC50 in a sub-nanomolar range.

In order to enhance the binding affinity of G12, we utilized in parallel two strategies: avidity enhancement by trivalent tandem repeats VHHx3-Fc protein construction and affinity maturation of G12 antibody by CDR1 and CDR2 shuffling.

### Affinity engineering of G12 binder

Affinity maturation is a well-established approach for antibody binding properties enhancement (20). In the absence of VHH-RBD complex structure, there are 2 options left to choose between: error-prone PCR and CDR shuffling. CDR3 contributes the most in VHH interactions with antigens, so we decided to re-randomize CDR1 and CDR2 loops not to compromise the affinity and specificity that we already had.

In our VHH4_DD library, unique restriction site was incorporated after the CDR2 region in order to provide the possibility of FR1-CDR2 region randomization by simple restriction\ligation steps. *E.coli* colonies harboring the VHH4_DD library were scrapped from 2 large 200 mm plates and were used for the phagemids pool preparation. FR1-CDR2 region from this pool was subcloned into G12 VHH for the secondary library formation. The resulting diversity of the secondary library was measured by the transformants counting after the electroporation and amounted to 10^8^ clones.

Three rounds of selection were performed with the biotinylated RBD concentration decreasing from 50 nM to 1 nM. After the 3rd round, monoclonal ELISA revealed that 15 out of 16 clones bind RBD with >10 signal-to-noise ratio (**Figure S2**). Sequence alignment showed a single sequence H7 prevalence which was presented in 12 from the 15 sequences. For the affinity measurements H7 clone was reformatted in the VHH-Fc fusion format and purified from the transiently transfected HEK293 FreeStyle supernatant (**Figure S3A**).

**Figure 6:**
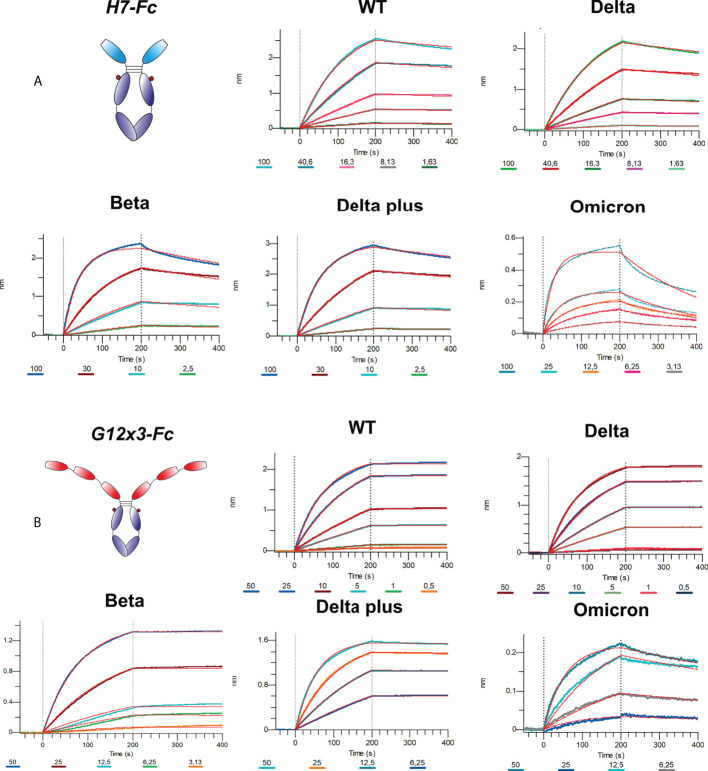
Multipoint BLI of H7-Fc **(A)** and G12x3-Fc **(B)** binders to SARS-CoV-2 VoC RBDs.

The binding analysis showed a 5.57-fold affinity improvement for H7 with the 4.4 nM *K_D_* value for the interaction with WT SARS-CoV-2 variant. We also performed extended binding studies with the emerging VoC RBDs – Delta, Delta plus, Beta and Omicron BA.1 (**Figure 6A**). An irrelevant VHH-IgG1 was used as an isotype negative control. This antibody was generated on the same scaffold against heavily-glycosylated antigen (16).

It was revealed that VoC mutations slightly affect the affinity of H7 to different RBDs and its *K_D_* value remains in the single-digit nanomolar range (**Table 1**). The unspecific binding of VHH-IgG1 to RBD variants was neglectable in comparison with RBD-specific samples (**Figure S4**).

**Table 1:** Antibodies binding activity against RBD variants; related to **Figure 6**.

	G12x3-Fc				H7-Fc			
	*K_D_* (M)	*k_on_* (1/Ms)	*k_off_* (1/s)	R^2^	*K_D_* (M)	*k_on_* (1/Ms)	*k_off_* (1/s)	R^2^
**WT**	<1x10^-12^	2.624x10^5^	<1x10^-7^	0.9996	4.485x10^-9^	8.937x10^4^	4.008x10^-4^	0.9991
**Delta**	<1x10^-12^	2.413x10^5^	<1x10^-7^	0.9996	7.722x10^-9^	7.213x10^4^	5.570x10^-4^	0.9995
**Delta+**	1.562x10^-10^	3.688x10^5^	5.760x10^-5^	0.9993	3.414x10^-9^	1.634x10^5^	5.579x10^-4^	0.9995
**Beta**	<1x10^-12^	2.538x10^5^	1.226x10^-7^	0.9983	3.722x10^-9^	2.535x10^5^	9.435x10^-4^	0.9976
**Omicron BA.1**	2.968x10^-9^	3.571x10^5^	1.060x10^-3^	0.9944	9.493x10^-9^	4.342x10^5^	4.122x10^-3^	0.9883

Considering the broad binding spectrum against SARS-CoV-2 variants, it was decided to improve its binding properties and a neutralization potency by another approach: avidity enhancement.

### Avidity engineering of G12 binder

One of the remarkable features of the VHH binding domains is their ability to be assembled into multivalent structures with the non-linear increase of binding properties (21). In addition to the expected avidity effect, it was assumed that linked VHH domains could bridge the distance between different RBDs on the same S-protein, but not between different S-proteins because of the inter-S-protein distance on the viral surface (22). It allows us to assume superiority of the tandem VHH variant in a live virus neutralization test.

A multivalent trimeric clone G12x3-Fc was constructed by fusion of three G12 sequences through flexible linkers L1 (GGGGSGGGGSGGGGS) and L2 (GGGGSGGGGSSGGGS) with the Fc domain of IgG1 protein. The resulting protein was transiently expressed in HEK293 FreeStyle and purified to the homogenous state (**Figure S3B**). Expression level decreased insignificantly from 15 mg\l to 12 mg\l.

Affinity measurements were performed by BLI on Octet R2 with the use of WT and VoC RBDs (**Figure 6B**). Kinetic data comparison with the affinity maturated H7-Fc protein revealed a 2-fold increase of *k_on_* values and more than 100-fold increase of *k_off_* values for WT, Beta, Delta and Delta plus variants (**Table 1**). Surprisingly, G12x3-Fc binding to Omicron BA.1 increased unproportionally only to 3 nM.

We also engineered G12x2-Fc tandem repeat fusion protein with the affinity to Delta and Beta variants of 0.6 nM and 1.6 nM, respectively (**Figure S5**). It was considerably less than the G12x3-Fc picomolar affinity, therefore, this clone was not considered further.

**Figure 7:**
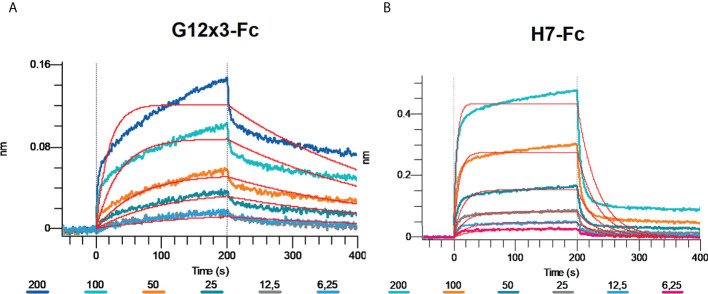
Multipoint Biolayer Interferometry (BLI) of G12x3-Fc binder **(A)** and H7-Fc binder **(B)** to SARS-CoV-2 Omicron BA.4/BA.5.

Omicron BA.4\BA.5 variants were the latest challenge for antibodies developed against WT SARS-CoV-2. Even most of the Omicron-specific antibodies raised previously against BA.1 could not effectively neutralize BA.4\5 (6, 23). We tested G12x3-Fc and H7-Fc binding to BA.4\5 variant by means of BLI (**Figure 7**).

Biphasic interaction mode didn’t allow us to calculate *K_D_* precisely, but from the interaction sensogram it is obvious that BA.4\5 RBD mutation significantly reduced antibodies binding that results in a fast *k_off_*. For further studies of BA.4\5 binding, structural information is needed that can suggest amino acid spots for the site-direction mutagenesis and secondary libraries creation.

Nevertheless, the most important criterion for the SARS-CoV-2 antibody potency is its neutralization, quantified by the inhibitory concentration (IC) values (e.g. IC50).

### Neutralization assay

The state-of-the-art method of determining the neutralization potential of antibodies is the plaque reduction neutralization test (PRNT). In this method, Vero E6 cells are infected with different variants of SARS-CoV-2 in the presence of serially diluted antibodies (24).

We used PRNT to determine if the H7-Fc and G12x3-Fc proteins were able to neutralize B.1.1.1, Delta and Omicron BA.1 SARS-CoV-2 virus variants. The neutralization IC50 potencies of these antibodies are shown in the **Table 2**.

**Table 2:** Neutralizing activity (PRNT) of top antibody candidates in comparison with the ACE2-Fc protein.

	IC50, ng\ml, SARS-CoV-2 variants		
	D614G	Delta	Omicron BA.1
**H7-Fc**	133.8	12.3	106
**G12x3-Fc**	13.1	0.9	9.6
**ACE2-Fc**	1664.1	52.1	391

The irrelevant VHH-IgG1 (B5-Fc) was used as a negative control for PRNT in order to exclude a possible influence of Fc-domain or VHH-scaffold on the results of the infection (**Table S2**). As the results of virus neutralization assays could vary from test to test in various laboratories, we included ACE2-Fc protein with the well-known IC50 value for different variants. H7-Fc antibody neutralized SARS-CoV-2 strains with an IC50 in a 12.3-133.8 ng\ml range. Encouragingly, the Omicron BA.1 variant could not escape the neutralization. G12x3-Fc binder showed a 10-fold decrease of IC50 values with 13.1 ng\ml, 0.9 ng\ml, and 9.6 ng\ml for B.1.1.1, Delta and Omicron BA.1, respectively.

## Discussion

Monoclonal antibodies due to their immediate virus neutralization properties represent a promising strategy for SARS-COV-2 treatment (25). Despite the decrease of a number of patients with severe cases in 2022, the people who have immunosuppression, including transplant recipients, those who have cancer, advanced or untreated HIV and autoimmune disorders, as well as the patients from high risk groups (older age>65, obesity or being overweight, pregnancy, chronic kidney disease, diabetes mellitus, cardiovascular disease, hypertension, chronic lung disease, etc.) still need the antibody treatment and pre- or post-exposure prophylaxis (5).

At the time of this manuscript preparation, the Omicron variants have become the dominant strain due to the highest transmissibility and the possibility to evade humoral immune response induced by all the major vaccines (26). Most of FDA approved therapeutic monoclonal antibodies including REGN10933, REG10987, LY-CoV555, LY-CoV016, AZD8895, and AZD1061 were found to be ineffective against the Omicron BA.1/BA.2/BA.4 and BA.5 variants, while only AZD7442 (Tixagevimab–Cilgavimab), VIR-7831 (Sotrovimab) and LY-CoV1404 (Bebtelovimab) were effective (7, 27–29). Moreover, it was suggested that Sotrovimab and other anti-Omicron antibodies could drive SARS-CoV-2 escape in immunocompromised patients (30). A wide variety of available antibody treatment options, including cocktails, or monoclonal antibodies rotation, could alleviate this problem.

In this work, we explored the possibility of promising VHH binders with the desired properties isolation from the novel synthetic VHH library. Such approach was proven to be successful by Seeger’s Sybodies libraries (31) and Kossiakoff’s synthetic high-performance Fab library (9), but with no bias towards variants of concern.

It was observed that the size of the library is strongly correlated with the affinity of isolated binders (32). However, it is crucial to maintain a high functional diversity reducing the proportion of clones incapable of affinity recognition and with poor developability properties (33).

Humanized nanobody h-NbBCII10_FGLA_ was used as a template for Kunkel mutagenesis for amino acid diversity introduction. Kunkel mutagenesis allows the library construction in a single step using a pool of randomized oligos without PCR overlapping or ligation stages (34). However, original non-mutated ssDNA does not eliminated completely during Kunkel mutagenesis resulting in a high level of a wild-type antibody in a library. It was observed that only a portion of the library’s clones have all CDRs randomized. All this leads to no more than 50% efficiency of the mutagenesis (11).

In order to overcome the above-mentioned issues, RCA of Kunkel mutagenesis-generated CCC-dsDNA was performed (12). RCA could reduce a template background by selective amplification of a newly synthesized mutated strand of DNA in heteroduplex after Kunkel and simultaneously enrich the transformable DNA. As a result, we constructed a large highly diverse library in 8 electroporation reactions, which is time- and labor-saving in comparison with the hundreds of electroporations for competing approach of library assembly by PCR and restriction\ligation (35). The resulted size of 1.2x10^11^ clones and more than 86% diversity puts it among the largest and most diverse phage display libraries (36, 37). The large 3.38x10^10^ transformants humanized VHH library reported recently was developed by means of 150 electroporation reactions (38). This library yielded a number of low-nanomolar RBD binders. However, that binding was abrogated by the VoC mutations which proves the necessity of using not only large VHH libraries, but also sophisticated screening technics for potent VHH isolation. Another 10^10^ transformants humanized nanobody library was constructed and screened against SARS-CoV-2 S–protein (39). Despite the high affinities of lead VHH candidates (low-nanomolar range), IC50 values were very moderate in comparison with the EUA-approved anti-SARS-CoV-2 binders. A more than 10^10^ diverse library based on a human VHH DP-47 scaffold was used for SARS-CoV-2 VHH generation by using a bead-based biopanning strategy by Twist Bioscience (8). A tremendous number of unique VHH binders were characterized resulting in best binder identification with the neutralization IC50 value <160 ng\ml.

As a proof-of-work, our objective was to generate a number of SARS-CoV-2 neutralization binders that could tolerate most of the VoC mutations by selection strategy modification. It was previously reported that the depletion of anti-RBD antibodies in convalescent patient sera results in the loss of >90% neutralizing activity of these sera against SARS-CoV-2 (40). It suggests that RBD binding by IgGs is the main and the most efficient mechanism of SARS-CoV-2 neutralization. It was also discovered, that RBD is the most potent immunogen protein for *Llama* spp. immunization for VHH generation in comparison with the S-protein or S1-subunit (41).

The small size and single-domain nature of VHH allow them to bind epitopes that are not available for conventional antibodies, in particular, concave epitopes such as grooves and clefts (42). Synthetic origin of the library also contributes to an increase in the possible diversity of epitopes compared to human antibodies.

During the first round we used the whole spike protein trimer as an immunogen to guide the selection towards the selection of antibodies against epitopes located within the RBD in the correct conformation of the intact spike protein trimer. For every subsequent round a different RBD variant –Wuhan-Hu-1 (WT), B.1.359 (Beta), B.1.617.2 (Delta) and B.1.1.529 (Omicron) as an antigen was used. Besides, negative selection using RBD-ACE2 complexes was performed in order to deplete binders that do not interfere with RBD-ACE2 binding. This is one of the key advantages of synthetic libraries: a precise control under the selection pressure, including negative selection and antigens rotation.

A panel of RBD specific VHH was selected with a picomolar and nanomolar binders among them. It was revealed that all binders have different sequences with CDR3 lengths of 9 and 13 amino acids. Despite the fact that the pool of libraries contains sub-libraries with CDR3 lengths of 9, 13, 15, and 18 amino acids, the longest CDR3 sequences were not enriched during the selection. Presumably shorter CDR3 is a more favorable paratope shape, which is more suitable for the binding on the RBD-ACE2 interface.

The potent antibody G12-Fc was isolated with the 20 nM affinities against WT and Delta variants. Two strategies not exceeding 3 weeks in length were tested to boost its potency.

In the absence of prior structural investigation, it was decided to perform affinity maturation by a blind CDR1 and CDR2 randomization. During the library construction step, we have included restriction sites flanking CDR1-CDR2 region in order to clone these CDRs diversity in selected candidates’ sequences.

Isolated from the secondary affinity maturated library clone H7 demonstrated the 5.57-fold affinity improvement with the 4.4 nM *K_D_* value for the interaction with WT SARS-CoV-2 variant. Its affinities against Delta, Delta plus, Beta, and Omicron BA.1 variants were 7.2 nM, 3.4 nM, 3.7 nM, and 9.5 nM, respectively.

It was suggested that the H7 clone belongs to the most promising class 3 or class 4 binders that prevent the Omicron escape (43). Nevertheless, affinity improvement of H7 was quite low in comparison with successful cases of affinity increasing by more than 2 orders of magnitude (44). A modest affinity enhancement could be explained by the non-significant role of CDR1 and CDR2 loops of G12 antibody in the interaction with RBD. This is not an uncommon situation for single-domain antibodies: to interact predominantly or even solely by the CDR3 hypervariable loop (45).

A multivalent trimeric clone G12x3-Fc was constructed with superior to G12-Fc and H7-Fc antibodies binding properties. *K_D_* values in a picomolar range for WT, Beta, Delta, and Delta plus variants put this antibody among the tightest RBD binders (46).

We investigated the neutralization activity of H7-Fc and G12x3-Fc antibodies using the plaque reduction neutralization test against live SARS-CoV-2 VoCs. It was elucidated that both of the antibodies efficiently block the virus entry into cells even regardless of the Omicron BA.1 escape mutations.

Despite the fact that the BA.4\5 variants were not included in the antigen’s rotation scheme during the selection, G12x3-Fc and H7-Fc binders retain binding to them. However, faster off-rate led to affinity, decreasing thus the applicability of these binders as anti-BA.4\5 agents that require further investigation.

Nevertheless, G12x3-Fc neutralization potency is not inferior to most of the SARS-CoV-2 binders approved to date, which proves the power of large synthetic antibody libraries and sophisticated selection techniques for cost-time effective antibodies with therapeutic potential isolation (47).

In conclusion, the antibody discovery workflow described herein represents an attractive alternative to the described methods of VHH generation by lama immunization or synthetic library development. Rolling-cycle amplification of the randomized DNA-pool that was generated by Kunkel mutagenesis allowed us to generate >10^11^ diversity humanized VHH phage display library. Such size and diversity puts it among the most diverse phage display libraries to date (48). The depletion panning strategy removed the binders targeting outside the receptor binding motif (RBM) resulting in neutralization antibodies generation. Changing the RBD variant in each round of the selection allowed this neutralization potential to be extended to a wide range of SARS-CoV-2 variants of concern.

The antibodies described in this report could expand the current landscape of therapeutic COVID-19 antibodies, as well as could be used for elucidation and clarification of the structural mechanisms of virus neutralization. As the synthetic VHH library and the selection strategy described above significantly differs from the natural immune response, it could lead to more diverse paratope space and result in more promising binders not only for SARS-CoV-2, but also for other antigens with the high mutation rate.

## Data availability statement

The original contributions presented in the study are included in the article/**Supplementary Material**. Further inquiries can be directed to the corresponding author.

## Author contributions

Conception and design of the experiments: DD and MK. Synthetic antibody libraries assembly: DD and AMe. Phage display and affinity maturation: DD. Cloning, production, purification and characterization of VHH and their Fc-fusions: MS, AK and SD. Production and purification of recombinant RBD and ACE2 proteins: AK and SD. BLI kinetic measurements: DD. SARS-CoV-2 neutralization tests: AMi and SS. Data analysis and interpretation, figures preparation: DD and MS. Drafting the manuscript: DD. All authors contributed to the article and approved the submitted version.

## Funding

The authors declare that this study received funding from Immunofusion. The funder was not involved in the study design, collection, analysis, interpretation of data, the writing of this article or the decision to submit it for publication.

## Conflict of interest

Authors MK, AMi and AMe are employees of UAB Imunovakcina and LLC Immunofusion. DD is a member of the Scientific Advisory Board of Imunovakcina. DD and MS have a pending patent application for the RBD-targeted antibodies from this study.

The remaining authors declare that the research was conducted in the absence of any commercial or financial relationships that could be constructed as a potential conflict of interest.

## Publisher’s note

All claims expressed in this article are solely those of the authors and do not necessarily represent those of their affiliated organizations, or those of the publisher, the editors and the reviewers. Any product that may be evaluated in this article, or claim that may be made by its manufacturer, is not guaranteed or endorsed by the publisher.
