# Supplementary material for: Isolation of an escape-resistant SARS-CoV-2 neutralizing nanobody from a novel synthetic nanobody library
Source: Front Immunol. 2022 Sep 16;13:965446. doi: 10.3389/fimmu.2022.965446 (PMC9524272; doi:10.3389/fimmu.2022.965446)
Supplement: Supplementary file 1 [file DataSheet_1.pdf]

Supplementary Material

Figure S1. Conversion of dU-ssDNA (5) to CCC-dsDNA (1-4) by Kunkel mutagenesis. 1-4 lanes stand for different sub-library synthesis. A smaller band of 1-4 lanes is CCC-dsDNA – covalently closed circular dsDNA, while larger bands are nicked and strand-displaced DNA.

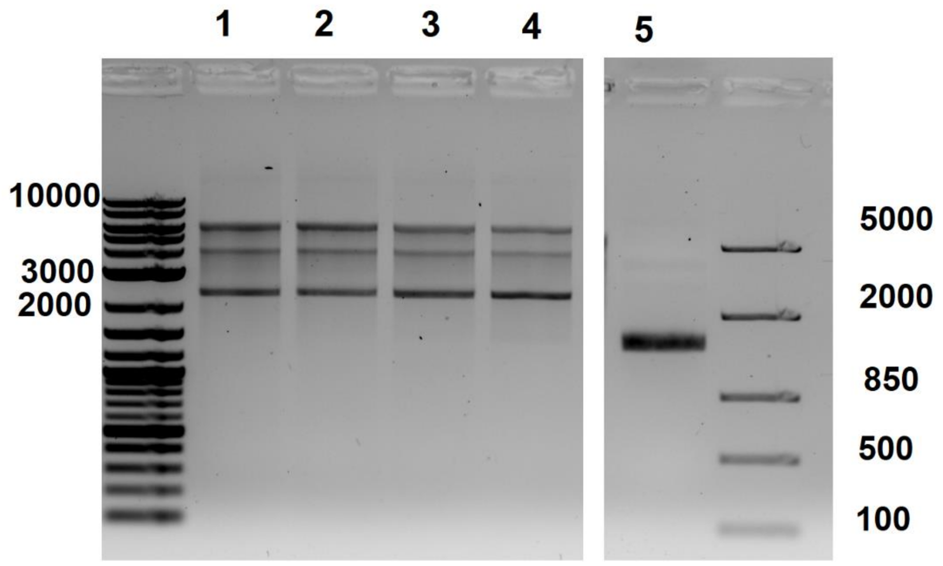

Figure S2. Monoclonal ELISA of selected VHH clones from the affinity maturated secondary library. Dashed red line – S\N threshold.

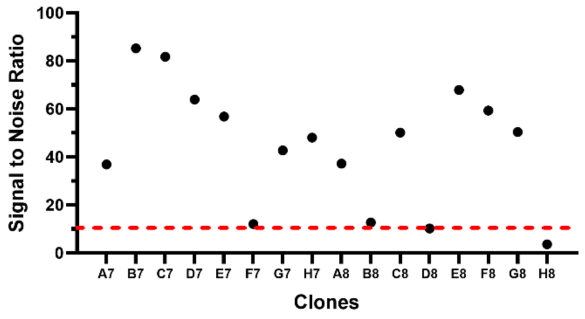

Figure S3. MALDI-TOF spectrum of purified G12x3-Fc (A) and H7-Fc (B) proteins. Insert: 12% SDS-PAGE reducing electrophoreses.

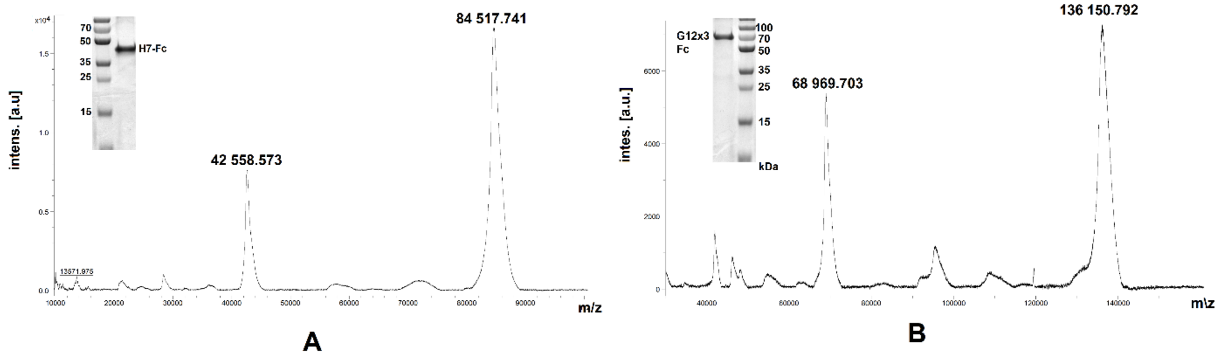

Figure S4. Raw data of the Octet analysis of 100 nM irrelevant VHH-IgG1 binding to RBD variants (baseline, RBD immobilization, baseline, VHH-IgG1 association, VHH-IgG1 dissociation) (A). Insert (B): Reference-corrected VHH-IgG1 association and dissociation steps.

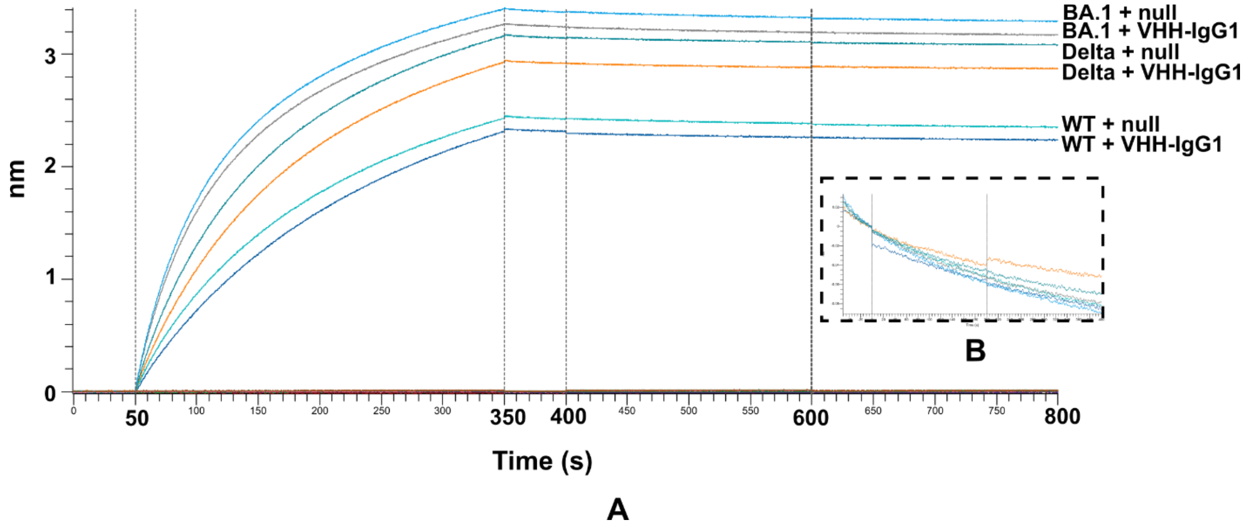

Figure S5. Multipoint BLI of G12x2-Fc binder to SARS-CoV-2 Delta (A) and Beta (B) RBDs

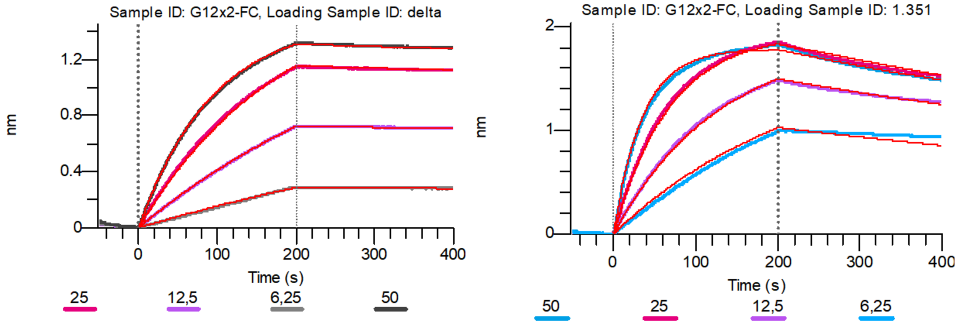

KD (M)	ka (1/Ms)	kdis (1/s)	Full R^2
6,106E-10	2,107E05	1,286E-04	0,9998

A

KD (M)	ka (1/Ms)	kdis (1/s)	Full R^2
1,643E-09	5,497E05	9,030E-04	0,9964

B

Table S1. List of oligonucleotides.

L452R_F	GTACCGGCTGTTCCGCAAGTCCAACCTGAAGC
L452R_R	CGGTAGTTGTAGTTGCCGCCGACTTTGCTGTC
T478K_F	AGCCTTGCAATGGCGTGGAAGGCTTCA
T478K_R	TGCTGCCGGCCTGGTAGATTTCGGTGC
E484K_F	GCTTCAACTGCTACTTCCCACTGC
E484K_R	CTTTCACGCCATTGCAAGGGGTGCT
K417N-F	GGACAGACAGGCAACATCGCCGATTACAAC
K417N-R	GTTGTAATCGGCGATGTTGCCTGTCTGTCC
N501Y-F	CTTCCAGCCTACATACGGCGTGGGCTAC
N501Y-R	GTAGCCCACGCCGTATGTAGGCTGGAAG
hACE2-r	CAATAGCGGCCGCGGAAACAGGGGGCTGG
hACE2-f	GAATACCATGGCCCAGTCCACCATTGAGGAACAGGC
VHHtri-NcoI-f	GCAATACCCATGGCCCAGGTTCAGCTGGTTGAAAGCGG
VHHtri-BamHI-r	CAAATAGGATCCTCCACCACCAGATCCTCCACCTCCGCTGCTAACGGTAAC
VHHtri-BamHI-f	CAATATGGATCCGGCGGAGGCGGCTCTCAGGTTCAGCTGG
VHHtri_XhoI-r	CAATACTCGAGCCTCCACCACCAGATCCTCCACCTCCAACGGTAACCAGG
VHHtri-XhoI-f	GAATATCTCGAGCGGAGGCGGCTCTCAGGTTCAGCTGG
VHHtri-NotI-r	GCGGCCGCGCTGCTAACGGTAACCAGGGTGC
pFUSE-FC-seq-r	CCAGGAGTTCAGGTGC

Table S2. VHH-Fc antibodies and ACE2-Fc PRNT raw data

	H7-Fc			G12x3-Fc			ACE2-Fc			B5-Fc	
**Concentration, ng\ml**	**B.1.1.1, pfu**										
10	1	0	0	0	0	0	7	4	7	11	10
1	2	0	1	0	0	0	8	8	10	10	9
0.1	5	5	7	0	0	0	8	10	10	7	8
0.01	9	10	9	7	6	9	8	9	10	9	8
0.001	8	8	9	10	10	9	9	8	9	10	11
0.0001	10	10	8	9	10	9	9	10	10	10	8
**Concentration, ng\ml**	**B.1.617.2, pfu**										
10	0	0	0	0	0	0	0	0	0	8	8
1	0	0	0	0	0	0	0	0	0	9	9
0.1	0	0	0	0	0	0	4	1	2	10	9
0.01	8	6	4	0	0	0	8	9	8	8	10
0.001	9	8	9	1	3	6	10	10	9	11	8
0.0001	8	9	9	8	9	9	8	8	9	8	9
**Concentration, ng\ml**	**B.1.1.529 BA.1, pfu**										
10	0	0	0	0	0	0	0	0	0	10	9
1	0	0	0	0	0	0	1	0	3	8	10
0.1	6	8	0	0	0	0	8	8	9	10	9
0.01	9	9	8	5	0	7	9	9	9	8	9
0.001	9	8	9	9	10	9	8	10	10	10	11
0.0001	7	10	11	8	8	10	8	10	8	10	8
